# A Novel Hybrid Runge Kutta Optimizer with Support Vector Machine on Gene Expression Data for Cancer Classification

**DOI:** 10.3390/diagnostics13091621

**Published:** 2023-05-03

**Authors:** Essam H. Houssein, Hager N. Hassan, Nagwan Abdel Samee, Mona M. Jamjoom

**Affiliations:** 1 Faculty of Computers and Information, Minia University, Minia 61519, Egypt; essam.halim@mu.edu.eg (E.H.H.);; 2 Department of Information Technology, College of Computer and Information Sciences, Princess Nourah bint Abdulrahman University, Riyadh 11671, Saudi Arabia; 3 Department of Computer Sciences, College of Computer and Information Sciences, Princess Nourah bint Abdulrahman University, Riyadh 11671, Saudi Arabia

**Keywords:** feature selection, Runge Kutta optimizer, microarray, gene expression, support vector machines, cancer classification, 68T99, 68U99

## Abstract

It is crucial to accurately categorize cancers using microarray data. Researchers have employed a variety of computational intelligence approaches to analyze gene expression data. It is believed that the most difficult part of the problem of cancer diagnosis is determining which genes are informative. Therefore, selecting genes to study as a starting point for cancer classification is common practice. We offer a novel approach that combines the Runge Kutta optimizer (RUN) with a support vector machine (SVM) as the classifier to select the significant genes in the detection of cancer tissues. As a means of dealing with the high dimensionality that characterizes microarray datasets, the preprocessing stage of the ReliefF method is implemented. The proposed RUN–SVM approach is tested on binary-class microarray datasets (Breast2 and Prostate) and multi-class microarray datasets in order to assess its efficacy (i.e., Brain Tumor1, Brain Tumor2, Breast3, and Lung Cancer). Based on the experimental results obtained from analyzing six different cancer gene expression datasets, the proposed RUN–SVM approach was found to statistically beat the other competing algorithms due to its innovative search technique.

## 1. Introduction

For the past few years, gene expression profiling has allowed scientists to simultaneously examine thousands of genes. As a result, significant knowledge has been gained regarding the processes that take place within cells. In situations involving cancer, having access to such particular information is helpful for both diagnosing the disease and evaluating the patient’s prognosis. Microarray data features such as low sample size, high noise levels, and high dimensionality persist in making gene selection a challenging task. Identifying the best candidates for gene classification is essential to resolving this problem. This method will not only cut down on computational costs, but it will also enable physicians to focus on a small subset of physiologically significant genes that are linked to specific cancers, allowing for the formulation of more cost-effective studies [1]. In addition, a method with a high degree of accuracy can aid people with cancer by facilitating early detection and the development of new treatments [2].

The “curse of dimensionality”, manifested by small sample sizes and the presence of genes that are unrelated to one another, plagues the microarray data [3,4,5]. Due to the presence of this condition, it is more challenging to classify the typical gene expression profile for any given sample. Gene selection has been applied to microarray gene expression analysis as a means of overcoming the “curse of dimensionality” by determining the subset of features that are most significant [6].

There are a few different ways to go about selecting genes, but they can all be broken down into three broad categories: embedding, wrapping, and filtering. Using statistical methods like minimum redundancy maximum relevance, information gain, and ReliefF, the filter strategy collects intrinsic gene properties in order to differentiate the targeted phenotypic class [7]. This approach is easy to implement, but it does not take into account the complex web of interactions between genes. In order to evaluate potential new feature gene subsets, the “wrapper” strategy [8] attempts to pick a tiny subset of the initial feature set, typically through the use of an induction algorithm. Wrapper methods typically outperform filters because they account for multivariate inter-correlation among genes. The optimum number of feature genes for a given classifier can be calculated automatically using the wrapper approach. The embedded method, like the wrapper strategy, permits the combination of many strategies for doing feature subset selection (Kahavi and John, 1997; [9]). Genetic algorithms (GAs) are commonly employed as the search engine for the feature subset in the embedding approach (GAs) [10], while other classification techniques, such as GA-SVM [11], GA-K closest neighbors (GA-KNN) [12], and so on, are used to pick the smallest feature set.

Selecting the significant genes from gene expression data has been implemented using the wellknown machine learning approaches, including Neural Networks (NN), support vector machines (SVMs), and K-nearest neighbor (KNN). There is some evidence that gene selection can improve the accuracy of sample classification [13]. According to studies, the “no free lunch theory” states that no single optimization strategy is generally accepted as providing a globally optimal solution to any given optimization problem [14]. Different practical engineering applications were used to evaluate the RUN algorithm’s efficacy in comparison to numerous metaheuristic algorithms. The RUN’s outcomes were encouraging and on par with previous competitions [15]. Consequently, the objective of this study is to select the genes in the gene expression profile that are the most predictive and beneficial by making use of RUN, a recently proposed optimization method. A preprocessing stage that uses the ReliefF filtering strategy is used to reduce the dimensionality of gene expression datasets and select the valuable genes from among these datasets. As they perform better than other classification methods [16], SVMs have become the most widely used classification method in the field of cancer research, most notably in the analysis of gene expression data [17,18]. in this study, SVM is used in a hybrid cancer classification algorithm. The article also compares the effectiveness of the RUN algorithm as a generation selection algorithm to the effectiveness of other well-known selection algorithms [19], such as the Whale Optimization Algorithm (WOA) [20], the Artificial Bee Colony (ABC) [21], the Harris Hawks Optimization (HHO) [22], the Hunger Games Search (HGS) [23], the Golden Jackal Optimization (GJO) [24], and Manta Ray Foraging Optimization (MRFO) [25]. To evaluate how well the suggested RUN—SVM strategy performs in comparison to existing selection algorithms, the experimental study makes use of six unique microarray datasets.

To emphasize the significance of this study, the following are the contributions that it makes:

- Here we provide RUN—SVM, a novel approach to classifying gene expression data that combines the SVM classifier with the RUN feature selection optimizer.
- Overcomes the “feature dimensionality curse” in order to eliminate unnecessary features and focus on the most informative ones when classifying cancers based on gene expression data.
- Assesses the effectiveness of the introduced classifier in binary and multiclass classification tasks using six benchmark datasets.
- Compares the capability and feasibility of the proposed classification algorithm to existing approaches through a comprehensive evaluation.

The remaining sections of the article are presented in the following order: The review of the available literature can be found in Section 2. In Section 3, we will go over the first stages of the classification methods that make use of ReliefF, RUN, and SVM. The RUN–SVM technique that has been suggested is discussed in Section 4. In Section 5, In Section, both the experimental design and the results of the experiments are described. Section 6 contains a summary of the findings as well as recommendations for further research.

## 2. Literature Review

A number of different gene selection optimization approaches have been presented in the literature as a way to improve the accuracy with which cancers are diagnosed. The following is a list of the most recent studies that have been conducted for the purpose of making suggestions as to which genes should be employed in the classification and diagnosis of cancer.

Li [1], proposed a strategy for gene selection that included a GA and a PSO with support vector machine (SVM) classification. Their proposed methodology was put to the test using data from microarray samples. The authors’ intention with their combined strategy was to steer the proposed method away from the local optimum. Using an improved binary PSO and an SVM classifier.

In order to analyze microarray datasets, it was suggested that combining SVM with the artificial bee colony approach might be beneficial [26]. The proposed model was implemented on the group of genes that were chosen in order to evaluate how well it worked. The high computing cost associated with the large dimensionality of the microarray data was a hindrance for all of the strategies, despite the fact that the proposed model performed better than earlier studies that used this methodology. The authors of this study conducted experiments with both genetic algorithm, and particle swarm optimization [2]. It was determined that the suggested approach of GA augmented with SVM is the best option. Instead, both of the techniques required training a classifier with every gene simultaneously due to the high complexity of gene expression datasets. Comparisons with sundry state-of-the-art techniques revealed outcomes that were competitive when measured against industry standards. In the majority of executions, they achieved results with a classification rate of 100% and few genes per subgroup (3 and 4). By combining IG and SVM, the authors of [27] achieved the best results possible for classifying cancers. The most informative and important genes were selected using IG from the original datasets, and then redundant genes were omitted using SVM. They then used the LIBSVM classifier to evaluate the quality of the collected informative genes [28]. For this investigation, the authors prioritized selecting the fewest genes while ignoring the pursuit of the highest accuracy.

In [29], the authors applied filter-, wrapper-, and correlation-based feature selector (CFS) techniques to the acute leukemia microarray dataset. These methods had been used with a number of ML algorithms, such as naive Bayes, decision tree (DT), and SVM. They demonstrated that by integrating several feature selection and classification techniques, relevant genes can be selected with strong certainty. This was the first paper to discuss the biological and computational evidence for zyxin’s role in leukaemogenesis.

In [30], The researchers used a 1-norm support vector machine with squared loss and a second predictor made up of an SVM and the nearest neighbor to classify cancers rapidly using NN. When it comes to assessment and storage that algorithm just needs a few genes. This, of course, ignores the question of how many genes were actually selected.

Mohamad et al., [31], suggested a near-optimal, small selection of the most beneficial genes relevant to cancer classification. The current rule for updating velocity and particle position was updated by the method of Mohamad et al.

Wrapper strategies combining the SVM classification method and the FireFly algorithm (FF-SVM) have been proposed as a means to classify cancer microarray gene expression profiles [32]. The experimental results showed that the suggested algorithm performed better in terms of classification accuracy and selected fewer genes than the other existing techniques. Also, the top five biologically significant genes associated with cancer across all ten datasets were determined.

The authors of [33] concentrated on the filter-based feature selection approach. The primary goals of the proposed effort were to shorten computation times and improve categorization and prediction accuracy. They suggested work that decreases the complexity of data collection as well as the redundancy between different features in order to accomplish this. They employed a score-based criterion fusion approach for feature selection that increased prediction accuracy and cut down on calculation time.

A hybrid model, SVM-mRMRe, was presented for gene selection by the authors of [34]. The most important genes from high-throughput microarrays have been extracted using the proposed model, which combines the feature selection approach, mRMRe, with the SVM classifier. The introduced algorithm was evaluated using eight benchmarking microarray datasets. Several aspects of the selected data set were analyzed by four different classifiers including NN, KNN, SVM, and random forest (RF). As demonstrated experimentally, the suggested approach enhances the current state of the art in cancer tissue classification while using fewer data.

In [35], a number of combinations of wrapping approaches (using NB and KNN with rank search, greedy stepwise, and best first) and ranking methods (using information gain [IG] with threshold of 1% and 5%) were employed for choosing the most crucial genes for microarray datasets. Brain cancer, CNS, breast cancer, and lung cancer datasets were among these datasets. Comparing the scenario where all features were employed with the experimental findings, all feature selection strategies consistently performed well (no feature selection methods). The NB with IG and wrapper (NB and best first) and the KNN with IG and wrapper (KNN and best first) acquired the best performance and defeated all other approaches out of the several techniques that were utilized.

A new hybrid feature selection technique was introduced in [36]. It involved five filters and a wrapper mechanism. It was discovered that the hybrid collection of individual filters has yielded better performance than the retrieved one of the single filter. The suggested method had been tried out and evaluated against well-known hybrid features. Using 10 benchmark micro-array datasets, the suggested strategy was evaluated and contrasted with cutting-edge approaches. Experimental results showed that the proposed strategy exhibited superior performance to state-of-the-art methods in regards to precision in classification.

## 3. The Methodology

Here, we provide an overview of the methods that would be employed in the proposed RUN—SVM classification technique. These methods include the SVM classification method, the RUN optimization method, and the ReliefF feature selection method for microarray data.

### 3.1. ReliefF Filter Method

The first Relief algorithm [37] has been developed further with the addition of ReliefF [38]. Selecting one instance at random from the data, the primary structure of Relief then finds its nearest neighbor from the other class as well as its nearest neighbor from the same class. Sampled instances’ attribute values are compared to those of their nearest neighbors in order to revise the relevance scores for each attribute. According to this line of thinking, a valuable feature should be able to distinguish between instances of several classes while maintaining the same value for instances of the same class. With its enhanced capabilities, ReliefF is better able to handle multiclass situations and input that is illegible or noisy. This methodology has global applicability, low bias, involves feature interaction, and has the potential to uncover local dependencies that are missed by other methodologies.

### 3.2. Runge Kutta Optimizer (RUN)

The goal of optimization algorithms is to enhance the efficiency with which they solve various optimization problems by striking a balance between exploiting and exploring the problem space. This is done in order to improve the efficiency with which they tackle various optimization problems. Iman Ahmadianfar and her colleagues recently presented the RUN, which is one example of such an optimization method [15]. This algorithm makes use of a specialized search mechanism that is founded on the Runge Kutta method, and RUN is an essential component of this algorithm. The second stage is termed solution quality enhancement, and its purpose is to increase the quality of the solutions produced for the researched optimization problem while simultaneously minimizing the hazards of becoming trapped in a locally optimal solution. The mathematical formulation of RUN is explained with the help of an example that can be found in the next subsection.

#### 3.2.1. Initialization

The decision variables $y_{n,v}$ of the optimization problem are initialized at random to begin the optimization process in RUN by:

(1) $$y_{n,v}=R_{min}+rand.\left(R_{max}-R_{min}\right)$$

where the lowest and highest ranges of the decision variable *v* of dimensions 1,2,…, and *M* are, respectively, $R_{min}$, and $R_{max}$.

#### 3.2.2. Solution Update

The well-known RUN mechanism is applied to the following equations in order to modify the solutions $y_{n+1}$ at each iteration:

(2) $$\begin{array}{cc} \; & if\;rand<0.5\\ \; & y_{n+1}=\left(y_{c}.SF.r.g+y_{c}\right)+SF.SM+\mu .randn.\left(y_{m}-y_{c}\right)\\ \; & else\\ \; & y_{n+1}=\left(y_{m}.SF.r.g+y_{m}\right)+SF.SM+\mu .randn.\left(y_{r1}-y_{r2}\right)\\ \; & end \end{array}$$

where $y_{r1}$, and $y_{r2}$ are solutions selected at random and μ is a typical distributed random number. randn is a random number in the range of [0, 1]. *r* is an integer number that may be 1 or −1. *g* is a random number in the range of [0, 2]. $x_{c}$ and $x_{m}$ are solutions around which the local search is performed in order to explore the promising regions in the search space. SF was used to achieve an appropriate balance between exploration and exploitation.

Additionally, using Δy, which is the position increment, and RUN coefficients k1 through k4, the subsequent equation can be used to calculate SF, an adaptable factor, and SM, a RUN-guided search method:

(3) $$SM=\frac{\Delta y\times y_{RK}}{6},y_{RK}=k1+2\times k2+2\times k3+k4$$

#### 3.2.3. Enhanced Solution Quality (ESQ)

Using the ESQ technique, RUN incorporates better solution quality, escape from the local optima, and quick convergence. In order to use this approach, three new solutions—$y_{new1}$, $y_{new2}$, and $y_{new3}$—will be created based on the equation shown below:

(4) $$y_{new1}=\beta \times y_{avg}+\left(1-\beta \right)\times y_{best},y_{avg}=\frac{y_{r1}+y_{r2}+y_{r3}}{3}$$

(5) $$\begin{array}{cc} \; & if\;rand<0.5\\ \; & if\;w<1\\ \; & y_{new2}=w.r.\left|\left(y_{new1}-y_{avg}\right)+randn\right|+y_{new1}\\ \; & else\\ \; & y_{new2}=w.r.\left|\left(u.y_{new1}-y_{avg}\right)+randn\right|+\left(y_{new1}-y_{avg}\right)\\ \; & end\\ \; & end \end{array}$$

(6) $$\begin{array}{cc} \; & if\;randn<w\\ \; & y_{new3}=SF.\left(\left(v.y_{b}-y_{new2}\right)+rand.y_{RK}\right)+\left(y_{new2}-rand.y_{new2}\right)\\ \; & end \end{array}$$

where the value of β is a random number between [0, 1]. $y_{best}$ is the global best solution throughout iterations. *r* is an integer that can be either 1, 0, or −1. $y_{b}$ is the best solution per iteration. A random number *v* is equal to 2 × randn. The random number *w* gets smaller as the number of iterations rises.

The pseudo-code steps of the RUN method is depicted in Algorithm 1.
**Algorithm 1** The RUN algorithm1:**Input:** Population size, Population initialization, and MaxIter of iterations.2:**Output:** The best Solution.3:Evaluate Objective Function using Equation (7).4:Calculate $y_{n+1}$ using Equation (2).5:**while** Stop condition not met **do**6:     **for** n = 1 ... N **do**7:           Evaluate Objective Function using Equation (7).8:           **if** <0.5 **then**9:                calculate $y_{new2}$ using Equation (5)10:               **if** $F(y_{n+1}<F\left(y_{new2}\right))$ **then**11:                   **if** rand < w **then**12:                         Calculate $y_{new3}$ using Equation (6).13:                         **if** $F\left(y_{n+1}\right)<F\left(y_{new3}\right)$ **then**14:                             $y_{n+1}=y_{new3}$15:                         **end if**16:                   **end if**17:               **else**18:                   $y_{n+1}=y_{new2}$19:               **end if**20:           **end if**21:     **end for**22:**end while**

### 3.3. Support Vector Machine (SVM)

In the realm of supervised machine learning, SVMs are a helpful tool for dealing with classification and regression issues [39]. Despite this, the majority of their applications are found in classification-related debates. It is difficult to find a linear classifier in the dataset that can differentiate between the different classes. The SVM, widely regarded as the only classification method capable of handling this situation, discovers a linear classifier model for categorizing the input data within an excess margin limit after mapping and modifying the input space to turn it into a high-dimensional space. This model is used to determine where the data falls within the space. When dealing with data that has a lot of dimensions, SVM performs the best as a classifier [40]. In light of this, an SVM classification technique is utilized in this investigation in order to assess how useful RUN is when applied to microarrays classification.

The results of a support vector machine are controlled by two variables. *C* regulates the compromise between accurate training-data classification and a consistently smooth resolution limit. Meanwhile, Γ represents the effect of a single training session. Algorithm 2 demonstrates the pseudo-code for the SVM model.
**Algorithm 2** The SVM algorithm1:**Input:** Test and training points.2:**Output:** Best fitness.3:Select the SVM’s expense factor *C* and Γ.4:**while** stop condition not satisfied **do**5:    For each data point, Apply the SVM training phase.6:    For testing data points, Apply the SVM classifying phase.7:**end while**8:**return fitness**

In order to partition the data into K separate sets, a statistical technique known as K-fold cross-validation is utilized. As a result, only the training subsets were employed at first; however, more recently, other subsets have been used to test or validate the training subsets. When testing the model, a pending subsample will be used for validating the data, and K-1 sets would be employed for training points.

## 4. The Proposed RUN-SVM Approach

**Figure 1:**
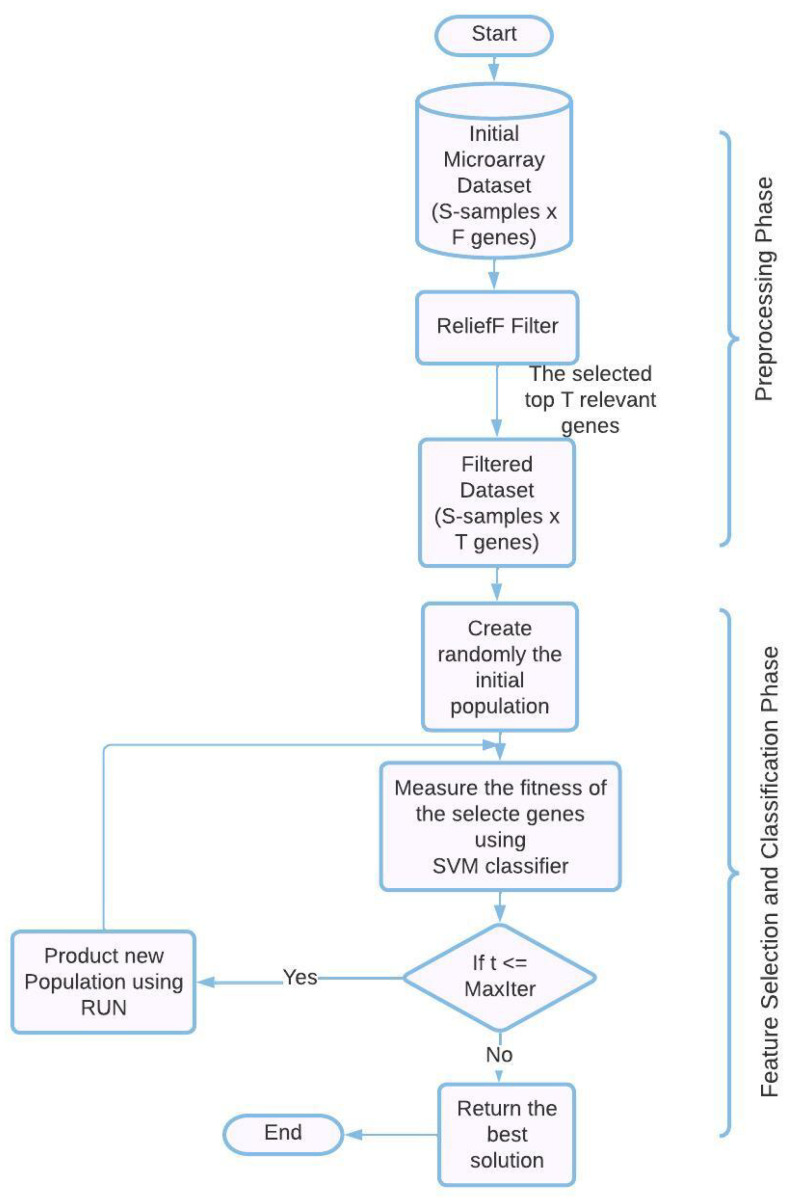
The proposed RUN-SVM approach.

The combination of ReliefF, RUN, and SVM into a single framework for the effective classification of microarray datasets is novel, as far as we are aware. Not only is RUN being used for the first time as a gene selection method for cancer gene expression datasets, but it is also being used in a novel way. Here, we offer an overview of the hybrid cancer classification method we’ve proposed for picking the most precise SVM-classified genes from cancer gene expression datasets. In order to offer a set of genes that are predictive as well as informative, the proposed RUN–SVM approach, which is depicted in Figure 1, includes two processes: (1) a preprocessing step, and (2) a feature selection (FS) and classification Step. These steps are displayed in order. It is also possible to evaluate the predictability and informability of the other gene selection strategies by using the same two steps of the RUN–SVM approach that we have presented.

### 4.1. Preprocessing Phase

Applying algorithms directly to microarray datasets is a real challenge due to the fact that these datasets are of a high dimension and contain thousands of genes that need to be filtered. As a result of this, using these kinds of datasets without first filtering them makes it difficult to accurately train the SVM classification algorithm. For its excellent performance, great efficiency, and widespread application in the field of resolving problems with huge dimensions, we decided to make use of the ReliefF algorithm in this particular piece of work. As a result, the first stage of the proposed RUN–SVM methodology is called ReliefF. It can be utilized for the purpose of removing redundant genes, which contribute to the background noise and lower the classification accuracy (for more information on ReliefF, see Section 3.1).

### 4.2. Feature Welection and Classification Phase

To pick the most important genes from the smaller pool of genes generated in the first stage, we use wrapper approaches like the RUN algorithm and the SVM. First, RUN generates a randomly selected initial population, which is then subjected to an evaluation based on a fitness function. When the FS and classification phase procedures have been completed, the criterion has been met, and the ideal solution has been created, one with the highest classification performance and the fewest informative genes. This guarantees a productive outcome from the procedure. The SVM classifier performs an evaluation of the RUN solutions through the repetition phase in order to gauge how effective they are. The fitness function is evaluated based on the percentage of samples that are correctly categorized in comparison to the total number of samples (for more information on RUN and SVM, see Section 3.2 and Section 3.3).

(7) $$fitness=\frac{C}{T}*100$$

where *C* indicates samples that were successfully classified, and *T* stands for the overall number of input samples.

The pseudo-code of the proposed RUN–SVM approach is demonstrated in Algorithm 3.
**Algorithm 3** Pseudo code of the proposed RUN-SVM approach1:**Input:** MaxIter, A Microarray Dataset, Population size, Population initialization2:**Output:** Best solution.3:Apply ReliefF filter on Dataset and generate a filtered dataset.4:Generate an initial population.5:Evaluate Objective Function using Equation (7).6:Calculate $y_{n+1}$ using Equation (2).7:**while** t< MaxIter **do**8:     **for** n = 1 ... N **do**9:           Evaluate Objective Function using Equation (7).10:           **if** rand<0.5 **then**11:               calculate $y_{new2}$ using Equation (5).12:               **if** $F(y_{n+1}<F\left(y_{new2}\right))$ **then**13:               **if** rand < w **then**14:                   Calculate $y_{new3}$ using Equation (6).15:                   **if** $F\left(y_{n+1}\right)<F\left(y_{new3}\right)$ **then**16:                       $y_{n+1}=y_{new3}$17:                   **end if**18:               **end if**19:           **else**20:               $y_{n+1}=y_{new2}$21:           **end if**22:        **end if**23:    **end for**24:**end while**

## 5. Experimental Evaluation and Discussion

### 5.1. Datasets and the Running Environment

In this investigation, we obtained the gene expression datasets by downloading them from a public database referred to as the GEMS Database. This database is a repository for gene expression data [41,42]. There are two types of gene expression datasets that have been downloaded: multi-class microarray datasets (i.e., Brain Tumor1, Brain Tumor2, Breast3, and Lung Cancer) and binary-class microarray datasets (Breast2 and Prostate). The dataset has been preprocessed to produce the gene expression matrix—S by F matrix, where S is the number of samples and F is the number of genes present in the given samples—which is used to represent the gene expression dataset. The six cancer gene expression datasets employed to evaluate the effectiveness of the proposed RUN–SVM approach are shown in Table 1. A computer with Microsoft Windows 8.1 with core i-5, 2.50 GHz, 64-bit, and 8 GB main memory was employed for the user testing environment, and Matlab R2015a served as the executable environment. Each of the 30 independent runs of the proposed RUN–SVM approach includes 30 search agents and 100 iterations.

### 5.2. Evaluation Metrics

The proposed RUN—SVM method was compared to the other approaches using four different measures of accuracy: best fitness function, standard deviation (STD), worst fitness function, and mean fitness function. In general, these are the four accuracy measurements that were used. The best fitness score at a run I is $fitness(y_{best})$.

1. **Mean:** is calculated by multiplying the number of times the algorithm is run by its fitness value (N).
2. **Best fitness function:** is a value that, after running the algorithm N times, outperforms all other best fitness values.
3. **Worst fitness function:** the smallest value for each maximum fitness metric that can be achieved by repeated iterations of the method N times
4. **The standard deviation (STD):** is a quantity that can be obtained by running the algorithm N times and measuring the amount of change in the fitness values. Data points with a smaller STD value show that they cluster tightly around the mean, while data points with a larger STD value suggest that they may deviate greatly from the mean.

### 5.3. Experimental Findings

The cross-validation method was implemented in this investigation in order to enhance classification performance, minimize bias in point selection for testing and training, and improve overall accuracy. Therefore, 10-fold cross-validation was utilized during the process of training the SVM classifier. In addition, the ReliefF filter and SVM classifier-improved comparison algorithms were each subjected to 30 separate runs with each run consisting of 100 iterations.

#### 5.3.1. Statistical Results Analysis

According to the results displayed in Table 2, Table 3 and Table 4, RUN ranks first in terms of best fitness values, worst fitness values, and mean in the Brain Tumor1, Brain Tumor2, and Breast3 datasets, respectively. Table 5 displays that the three fitness metrics with the highest rankings in Beast2 are RUN, MRFO, and HGS, with RUN being the gold standard. Table 6 displays that RUN and HGS have the highest fitness values in the Lung Cancer dataset, but RUN also has the lowest standard deviation. While HGS is rated highest for greatest fitness, Table 7 shows that RUN ranks highest for mean, worst, and standard deviation on the Prostate dataset.

#### 5.3.2. Convergence Behavior Analysis

**Figure 2:**
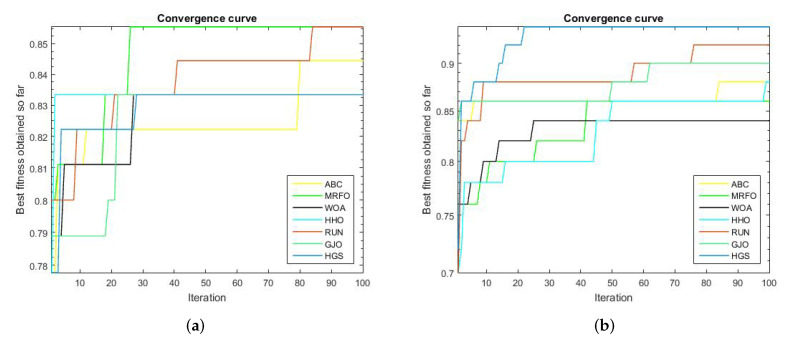

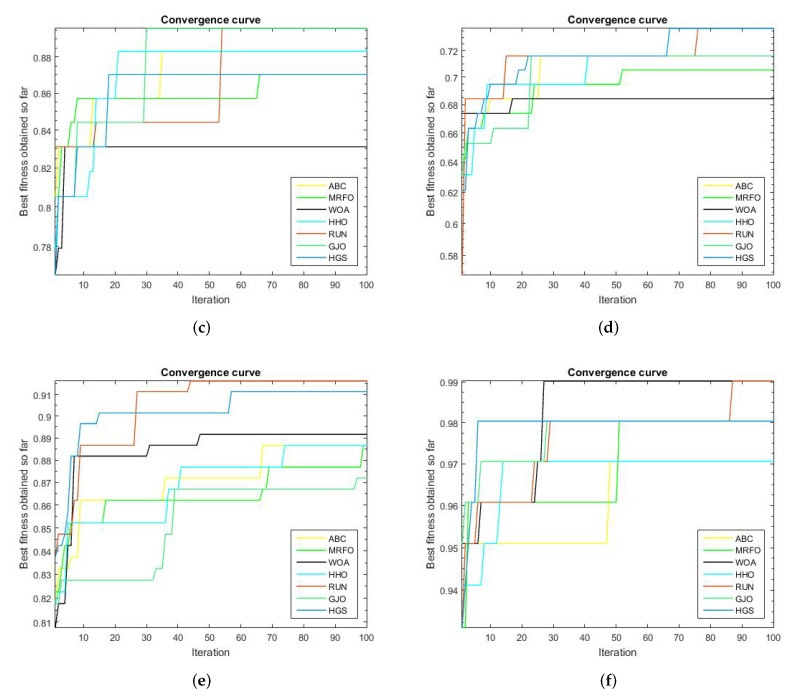
Convergence curves for the comparison algorithms. (**a**) Brain Tumor1 Convergence Curve. (**b**) Brain Tumor2 Convergence Curve. (**c**) Breast2 Convergence Curve. (**d**) Breast3 Convergence Curve. (**e**) Lung Cancer Convergence Curve. (**f**) Prostate Convergence Curve.

We compared the proposed RUN–SVM approach to some other popular optimization techniques and showed how it converges after 100 iterations. Based on the results shown in Figure 2a, the proposed RUN–SVM approach and the MRFO algorithm are the only ones that can be recognized to have the best accuracy for the Brain Tumor1 dataset. According to Figure 2c, only the proposed RUN–SVM technique and the GJO algorithm are able to achieve the maximum accuracy for the Breast2 dataset within the 100 iterations.

Figure 2d, shows that the introduced RUN–SVM algorithm, together with the HGS algorithm, are the only algorithms that can be said to attain the best performance in the classification of samples in the Breast3 dataset. In addition, Figure 2e, the proposed RUN–SVM approach is the sole algorithm to achieve the greatest accuracy for the Lung Cancer dataset. Only the WOA algorithm and the suggested RUN—SVM method have been shown to achieve the maximum precision on the Prostate database, as depicted in Figure 2f.

#### 5.3.3. Boxplot Behavior Analysis

**Figure 3:**
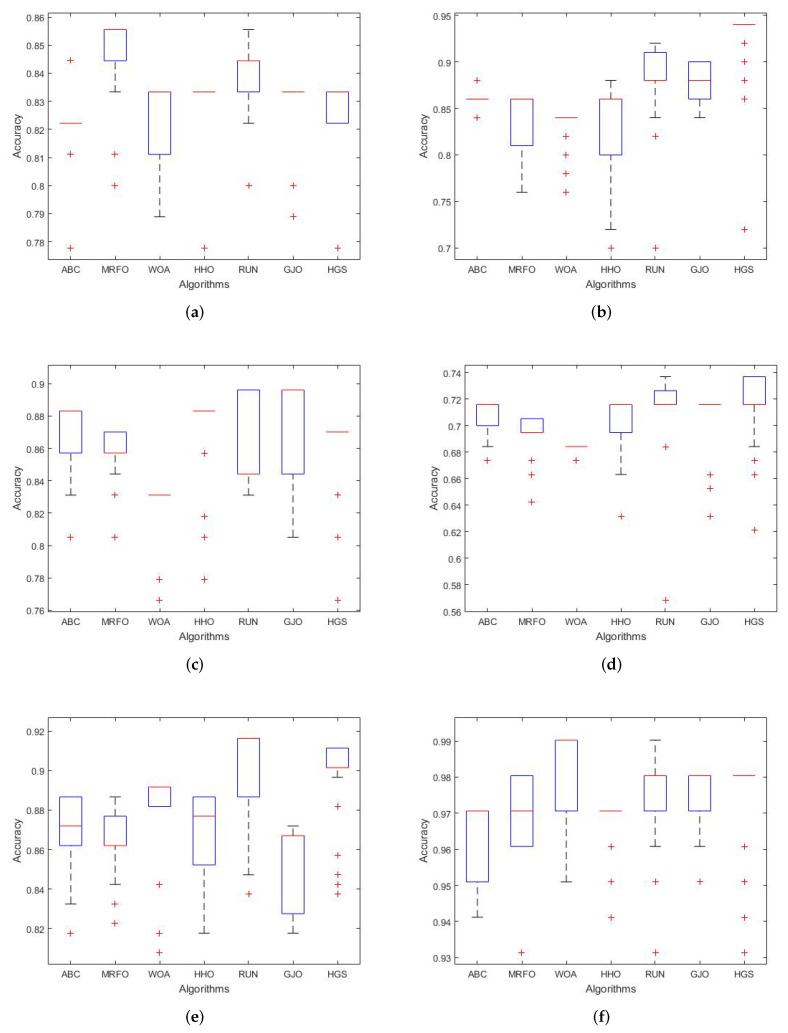
Boxplots curves for the comparison algorithms. (**a**) Brain Tumor1 Boxplot. (**b**) Brain Tumor2 Boxplot. (**c**) Breast2 Boxplot. (**d**) Breast3 Boxplot. (**e**) Lung Cancer Boxplot. (**f**) Prostate Boxplot.

In this section, we draw attention to the results of the proposed RUN–SVM approach. The proposed RUN–SVM approach outperformed the competition in this evaluation, as is shown by the boxplot curves in Figure 3a–f.

### 5.4. Discussion

**Figure 4:**
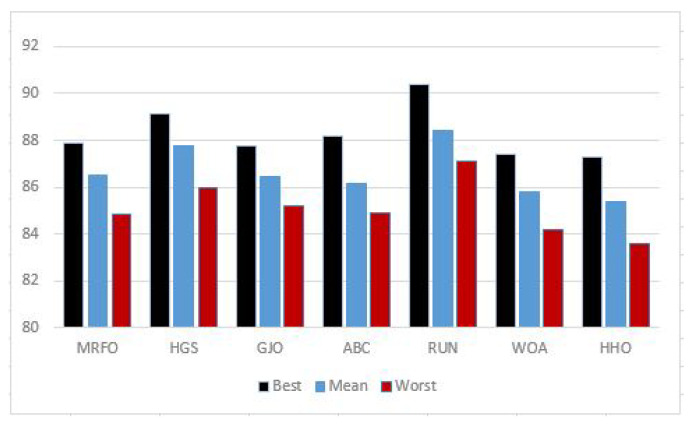
Average accuracy percentage.

The purpose of this section is to examine the numerous systems now in use for classifying cancers. The goal of this research is to propose a search strategy for the FS problem that takes into account the challenges posed by high-dimensional datasets. The research suggested using a ReliefF filtering approach for preprocessing and then combining RUN and SVM for the FS and classification stages. The effectiveness of the proposed RUN–SVM approach is demonstrated by experimental analysis and a comparison study. The following is a list of benefits that come with using our proposed RUN–SVM technique:

- The datasets that were utilized for the analysis of this study contain feature sizes that range anywhere from 4869 to 12,600 features, which makes for an excellent testing environment for an optimization method.
- Any chance for improving RUN–SVM can easily be implemented due to its straightforward architecture.
- Figure 4 shows how RUN–SVM outperforms other optimization algorithms in terms of classifying cancer.

On the other hand, the proposed RUN–SVM method offers certain restrictions, and these are listed below.

- Due to the fact that RUN–SVM uses a randomization-based optimization technique, the features it uses may vary from run to run. The feature subset picked in one run may not be present in another.
- As most genes are related to one another either directly or indirectly, microarray data processing is quite complex.
- The limited number of samples available in microarray datasets, the presence of noise and genes that are not relevant to the study, and the “curse of dimensionality” all add to the difficulty of classifying a particular sample.

## 6. Conclusions and Future Work

Using the Runge Kutta optimizer (RUN) approach, this investigation presents an improved way for choosing significant genes from cancer microarray patterns. The first primary goal was to pick the genes from the microarray datasets that were the most relevant and helpful with the least amount of effort, and the second primary goal was to select the genes that were the most accurate with the least amount of effort. In this paper, a wrapper gene selection approach called RUN—SVM is proposed. RUN—SVM combines the RUN algorithm with the support vector machine (SVM) classifier in order to identify the most relevant genes from a small dataset after removing redundant genes and selecting related genes from gene expression datasets. Six standard microarray datasets were used to evaluate the proposed RUN–SVM approach, namely Brain Tumor1, Brain Tumor2, Breast2, Breast3, Lung Cancer, and Prostate. The findings of the experiments showed that the RUN-SVM methodology obtains the highest level of accuracy in comparison to other comparative methodologies, such as HHO, MRFO, GJO, HGS, WOA, and ABC. The effectiveness of recent deep learning classifiers, such as transformer-based approaches and self-attention methods, could be examined for cancer classification using microarray datasets as part of future work that is associated with this research. Also, the performance of those classifiers could be evaluated in order to determine whether or not it would be enhanced by making use of the optimizer that was proposed in this study.

## Figures and Tables

**Table 1:** An overview of the datasets acquired by the microarrays.

Datasets	Genes	Samples	Classes
Brain Tumor1	5920	90	5
Brain Tumor2	10,368	50	4
Breast2	4869	77	2
Breast3	4869	95	3
Lung Cancer	12,600	203	5
Prostate	10,509	102	2

**Table 2:** Performance evaluation of the proposed algorithm on the Brain Tumor 1 dataset.

Algorithms	STD	Best	Mean	Worst
MRFO [25]	1.217	86.667	84.667	83.333
HGS [23]	0.4969	86.667	85.778	85.556
GJO [24]	1.7568	86.667	84.444	82.222
ABC [21]	0.9938	85.556	84	83.333
WOA [20]	0.9296	84.444	83.556	82.222
HHO [22]	1.4487	84.444	83.111	81.111
RUN-SVM	2.3040	90	86.222	84.444

**Table 3:** Performance evaluation of the proposed algorithm on the Brain Tumor 2 dataset.

Algorithms	STD	Best	Mean	Worst
MRFO [25]	1.7889	88	86.8	84
HGS [23]	1.673	92	90.4	88
GJO [24]	0.8944	90	88.4	88
ABC [21]	2.280	92	88.8	86
WOA [20]	2.1909	92	88.4	86
HHO [22]	2	86	84	82
RUN-SVM	2.280	94	90.8	88

**Table 4:** Performance evaluation of the proposed algorithm on the Breast 3 dataset.

Algorithms	STD	Best	Mean	Worst
MRFO [25]	1.289	72.632	71.579	69.474
HGS [23]	1.729	73.684	72.421	70.526
GJO [24]	1.3725	72.632	71.368	69.473
ABC [21]	0.471	72.632	71.789	71.579
WOA [20]	1.596	71.579	69.895	68.421
HHO [22]	2.025	74.737	71.789	69.474
RUN-SVM	2.306	76.842	73.053	71.579

**Table 5:** Performance evaluation of the proposed algorithm on the Breast 2 dataset.

Algorithms	STD	Best	Mean	Worst
MRFO [25]	1.693	90.91	89.351	87.013
HGS [23]	1.162	90.91	90.389	88.312
GJO [24]	1.087	88.117	87.273	85.714
ABC [21]	1.837	89.61	87.013	85.714
WOA [20]	1.299	88.312	87.013	85.714
HHO [22]	1.926	88.312	85.974	83.117
RUN-SVM	0.7113	90.91	90.13	89.61

**Table 6:** Performance evaluation of the proposed algorithm on the Lung Cancer dataset.

Algorithms	STD	Best	Mean	Worst
MRFO [25]	0.9603	90.148	88.867	88.177
HGS [23]	2.072	91.626	89.557	86.207
GJO [24]	1.155	90.148	88.669	87.685
ABC [21]	1.889	91.133	88.571	86.699
WOA [20]	2.013	89.163	87.586	84.729
HHO [22]	1.419	91.133	88.867	87.685
RUN-SVM	0.6033	91.626	91.133	90.148

**Table 7:** Performance evaluation of the proposed algorithm on the Prostate dataset.

Algorithms	STD	Best	Mean	Worst
MRFO [25]	0.820	99.02	97.843	97.059
HGS [23]	1.201	100	98.039	97.059
GJO [24]	0.537	99.02	98.431	98.04
ABC [21]	0.877	98.039	96.667	96.078
WOA [20]	0.438	99.02	98.235	98.04
HHO [22]	0.537	99.02	98.627	98.039
RUN-SVM	0	99.02	99.02	99.02

## Data Availability

Data sharing is not applicable to this article, as no datasets were generated or analyzed during the current study.
